# Association of metabolic score for insulin resistance with incident metabolic syndrome: a cohort study in middle-aged and older adult Chinese population

**DOI:** 10.3389/fpubh.2025.1453144

**Published:** 2025-02-18

**Authors:** Qiuling Zhang, Yushuang Wei, Shengzhu Huang, YeMei Mo, Boteng Yan, Xihui Jin, Mingjie Xu, Xiaoyou Mai, Chaoyan Tang, Haiyun Lan, Rongrong Liu, Mingli Li, Zengnan Mo, Wenchao Xie

**Affiliations:** ^1^The First People’s Hospital of Yulin, Yulin, Guangxi, China; ^2^School of Public Health, Guangxi Medical University, Nanning, Guangxi, China; ^3^Institute of Urology and Nephrology, First Affiliated Hospital of Guangxi Medical University, Guangxi Medical University, Nanning, Guangxi, China; ^4^Guangxi Key Laboratory for Genomic and Personalized Medicine, Guangxi Collaborative Innovation Center for Genomic and Personalized Medicine, Center for Genomic and Personalized Medicine, Guangxi Medical University, Nanning, Guangxi, China

**Keywords:** METS-IR index, metabolic syndrome, older adult, abdominal obesity, HDL-C (high density lipoprotein)

## Abstract

**Background:**

Recent studies suggest that the metabolic score for insulin resistance (MetS-IR) is an effective indicator of metabolic disorders. However, evidence on the relationship between MetS-IR and metabolic syndrome (MetS) among the Chinese middle-aged and older adult population is limited.

**Objective:**

This cohort study aims to assess the associations of MetS-IR levels with MetS risk and its components.

**Methods:**

Data used in this study from the National Basic Public Health Service Project Management System (2020–2023). Multivariable Cox proportional hazards model and restricted cubic spline (RCS) were employed to evaluate the associations of baseline MetS-IR levels with MetS risk and its components, receiver operating characteristic (ROC) curves were further utilized to assess the efficacy of MetS-IR in predicting the risk of MetS and its component.

**Results:**

Of 1,498 subjects without MetS at baseline, 392 incident MetS cases were observed during a median of 27.70 months of follow-up. The adjusted multivariable Cox regression analysis indicated an elevated 15% risk of developing MetS for 1-SD increment of MetS-IR [hazard ratios (HRs) and 95% confidence intervals: 1.16 (1.13–1.18)]. Compared to the first tertile of MetS-IR, the HRs of the third tertile and second tertile were 6.31 (95% CI 4.55–8.76) and 2.72 (95% CI 1.92–3.85), respectively. Consistent findings were further detected across subgroups. Moreover, nonlinear associations were observed between MetS-IR and the risk of MetS, abdominal obesity, and reduced high-density lipoprotein concentration (HDL-C) (*P*_nonlinear_ < 0.01), with the cutoff of MetS-IR was 32.89. The area under the curve for MetS-IR in predicting MetS was 0.740 (95% CI 0.713–0.768), which was better than those of other indicators.

**Conclusion:**

Our cohort study indicates a positive nonlinear association between MetS-IR with incident MetS, abdominal obesity, and reduced HDL-C, but positive linear associations of MetS-IR and elevated blood pressure (BP), elevated fasting blood glucose (FBG), elevated triglycerides (TG) in middle-aged and older adult people, more studies are warranted to verify our findings.

## Introduction

MetS is a comprehensive state of systemic metabolic disruption and is commonly characterized as central obesity, insulin resistance, hypertension, dyslipidemia, and hyperglycemia (1). As China’s population rapidly aging, disability due to age-related diseases has become a substantial socio-economic burden (2, 3). MetS is one of the most prevalent chronic diseases in the older adult population, which directly increases the risk of cardiovascular disease, type 2 diabetes, and all-cause mortality (4, 5). In China, the prevalence of MetS was estimated to be 36.96% among the older adult population, which is significantly higher than the general population (6, 7) However, the definition and diagnostic criteria of MetS has been a controversial issue since the initial conceptualization raised in 1923 (8). Additionally, existing definitions are binary variables that insufficiently identify individuals at critical risk. Hence, our efforts are focused on discovering an efficient continuous biomarker to comprehensively evaluate the overall disease status. This initiative aims to ensure precise assessment of individual risks and widespread applicability.

Insulin resistance (IR) is considered the most plausible hypothesis for MetS pathophysiology (9, 10). Currently, the hyperinsulinemic-euglycemic clamp is commonly used in clinical practice to assess insulin resistance (11). However, this technique has several limitations due to cost and technical complexity (11, 12). The metabolic score for insulin resistance (MetS-IR) is an indicator that has been used widely as an indirect method for estimating insulin resistance. The calculation typically involves in the following parameters: fasting blood glucose (FBG), triglycerides (TG), body mass index (BMI), and high-density lipoprotein concentration (HDL-C) (13). Cumulative evidence has proved the role of MetS-IR on hypertension, type 2 diabetes mellitus and adverse cardiovascular events (14–17). The study in children found that MetS-IR correlates with MetS (18). A Thai study found that MetS-IR may be a valuable tool in predicting MetS in younger police personnel (19). These findings suggest that MetS-IR is not only associated with the presence of MetS but also may predict its onset. However, to date, only a few studies have assessed the potential role of MetS-IR in anticipating and managing MetS among middle-aged and older adult populations.

Herein, our study focuses on the middle-aged and older adult population to explore the relationships between MetS-IR with MetS and its components and further to assess the diagnostic efficacy of MetS-IR in identifying MetS using the ROC curve. Our study contributes to the early screening of MetS in middle-aged and older adult populations and provides a scientific reference for auxiliary clinical diagnosis.

## Methods

### Study design and population

**Figure 1:**
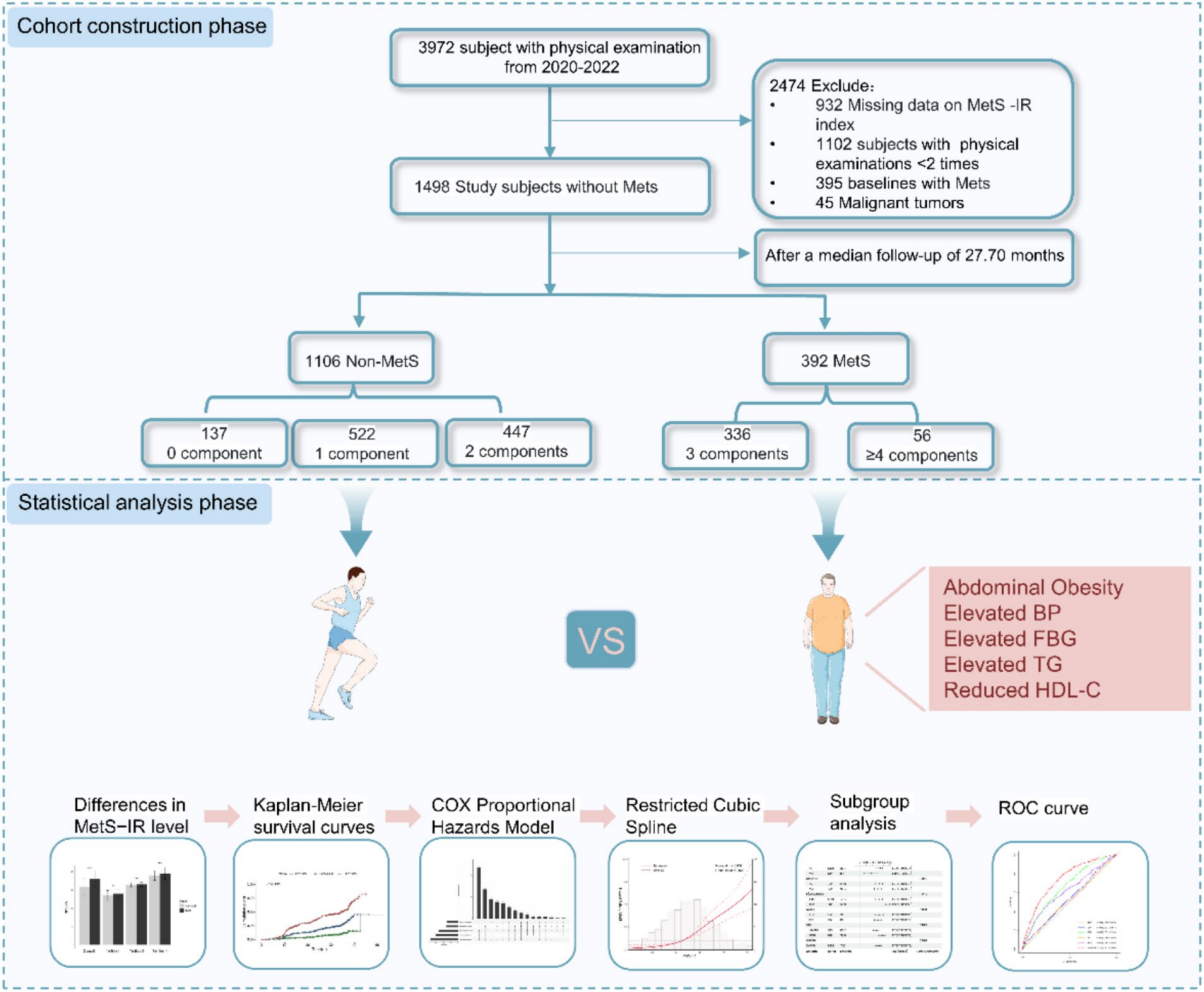
Study flowchart of subject selection. MetS–IR, metabolic score for insulin resistance; Mets, metabolic syndrome.

The data used in this study was obtained from the public health service (BPHS) management system. BPHS aims to provide primary health care services to the target population, including disease control, management of chronic disease, health promotion and education (20). The older adult people aged (65+) are eligible to receive complimentary healthcare services, including health management, health check-ups, and health guidance services. Participants under management have consented to the potential use of their health record data for scientific research purposes. These participants have annual follow-up assessments. We derived health record data between 2020 and 2022 from Wuliqiao Community Health Service Center of the First People’s Hospital of Yulin City, Guangxi. The inclusion criteria of the participants are as follows: (1) age ≥ 45 years old; (2) having physical examination data ≥2 times; (3) MetS has not been diagnosed at the first visit. We further excluded the participants with no baseline data available for FBG, TG, and those suffering from malignant tumors, autoimmune diseases, mental diseases. Finally, we included 1,498 subjects without MetS in the cohort study from 2020 to 2022. The flowchart for this study is shown in Figure 1.

### Measurement of baseline characteristics

Information on demographic characteristics [age, gender, ethnicity, waist circumference (WC), weight, height, etc.], exercise frequency (every day, more than once a week, occasionally, no exercise), smoking (yes, no), drinking (yes, no), disease history, and medication history were collected and updated annually by a face-to-face interview. BMI is calculated as weight (kg) divided by height squared (m^2^), a WHtR is for WC/height. Blood pressure was evaluated twice on the participant’s right arm using a validated electric BP monitor after at least 15 min of rest. The average of two measurements was documented as the individual’s BP.

Fasting blood samples were collected at the baseline visit to test serum white blood cells, FBG, TC, TG and HDL- C, low-density lipoprotein cholesterol (LDL-C) by local hospital.

### Definition of main variables

MetS-IR was calculated using the formula: (Ln (2*FBG + TG) *BMI)/(Ln (HDL–C)) (13). MetS were determined using the Chinese Diabetes Branch of the Chinese Medical Association (CDS2013) criteria. MetS was defined as the presence of no less than three risk factors as follows: (1) abdominal obesity: WC ≥ 90 cm (men), WC ≥ 85 cm (female), (2) Elevated BP: BP ≥ 130/85 mmHg and/or those who have been diagnosed and treated for hypertension, (3) Elevated fasting glucose: FBG ≥ 6.1 mmol/L or 2hPG ≥ 7.8 mmol/L and/or have been Diagnosis of diabetes and treatment, (4) Elevated triglycerides: TG ≥ 1.7 mmol/L, (5) Reduced HDL-C: HDL-C < 1.04 mmol/L.

### Statistical

Person time of follow-up was determined from the time at the baseline visit until the time at diagnose of MetS or the end of the study (December 2023). The baseline characteristics were described as frequencies and proportion for categorical variables, mean and standard deviation (SD) for continuous variables with normal distribution otherwise median and interquartile ranges for continuous variables with skewed distribution. To assess group differences, the Mann–Whitney U test was utilized for skewed distributed continuous variables, ANOVA for normally distributed continuous variables, and Chi-square tests for categorical variables. For the variable white blood cell count (WBC), which was missing for 196 participants (13.08%), we employed the multiple imputation method using the mice package in R, utilizing chained equations to handle the missing values.

The study first divided the MetS-IR variable into three groups according to tertile cut-points, then employed the Kaplan–Meier method to construct cumulative incidence rate survival curves for MetS events over time (months) across the three groups. The log-rank test was used to compare differences in the survival curves between the MetS-IR groups. Subsequently, multivariable-adjusted Cox proportional hazards model was performed to estimate the relationships between MetS-IR and MetS and associated components, adjustment covariates included gender, age, Exercise Frequency, Smoking, Alcohol use, WBC, antihypertensive medication, and antidiabetic medication. Further RCS was employed to explore potential nonlinear relationships between exposure and outcome. Additionally, we also conducted the interaction and subgroup analyses according to gender (male, female), age (≤65, >65), WHtR (≤0.5, >0.5), hypertension (yes, no), diabetes (yes, no), to assess the associations between MetS-IR and MetS. A sensitivity analysis was performed by excluding individuals with imputed data to reassess the relationship between MetS-IR and metabolic syndrome. Finally, ROC curve analysis was applied to evaluate the predictive performance of MetS-IR, SBP, DBP, BMI, TG, and FBG for MetS risk.

All analyses were performed using R statistical software version 4.3.0 and SPSS version 27.0 for Windows, and two-sided *p*-values < 0.05 were considered statistical significance.

## Results

### Baseline characteristics of the subjects according to MetS-IR categories

As shown in Tables 1, a total of 1,498 subjects without MetS at baseline from 2020 to 2022 were included in this cohort study, with a mean age of 69.76 ± 8.23 years. Most of them were mainly female (60.08%), non-smokers (96.53%), non-drinker (95.66%), and having exercise every day (89.95%). We finally observed 392 incident MetS cases during 3211.05 person-years of follow-up (27.70 months), and the incidence rate of Mets was 12.21 cases per 100 person-years for all subjects. Statistically significant differences were also observed in gender, age, DBP, WHtR, BMI, FBG, TC, TG, LDL-C, HDL-C, WBC, hypertension, diabetes, and antihypertensive medication across MetS-IR groups (*p* < 0.05).

**Table 1:** Characteristics of the cohort study subjects grouped by MetS-IR tertiles at baseline.

Variable	Overall (*N* = 1,498)	MetS-IR			*p*-value
		Tertile I (18.57–30.64)	Tertile II (30.64–34.82)	Tertile III (34.82–50.82)	
Gender (%)					<0.001
Male	598 (39.92)	164 (32.73)	201 (40.28)	233 (46.79)	
Female	900 (60.08)	337 (67.27)	298 (59.72)	265 (53.21)	
Age (yeas)	69.76 ± 8.23	71.06 ± 8.22	69.49 ± 8.19	68.73 ± 8.12	<0.001
SBP (mmHg)	130.95 ± 12.86	130.91 ± 11.66	131.58 ± 12.57	130.37 ± 14.21	0.572
DBP (mmHg)	79.62 ± 7.60	78.85 ± 7.59	80.01 ± 6.98	79.99 ± 8.14	0.002
Height (cm)	157.86 ± 8.06	156.51 ± 8.03	157.88 ± 7.88	159.20 ± 8.08	<0.001
Weight (cm)	57.86 ± 9.74	50.03 ± 6.84	57.77 ± 6.55	65.83 ± 8.39	<0.001
WC (cm)	84.74 ± 8.37	79.24 ± 7.05	84.58 ± 6.41	90.45 ± 7.51	<0.001
WHtR	0.53 (0.50 0.57)	0.50 (0.48 0.54)	0.53 (0.51 0.56)	0.56 (0.53 0.60)	<0.001
MetS-IR	32.75 ± 5.23	27.07 ± 2.70	32.80 ± 1.20	38.42 ± 3.00	<0.001
BMI (kg/m^2^)	23.14 ± 2.98	20.37 ± 1.88	23.13 ± 1.44	25.94 ± 2.35	<0.001
Exercise frequency (N,%)					0.732
Every day	1,346 (89.85)	451 (90.02)	455 (91.18)	440 (88.35)	
More than once a week	30 (2.00)	9 (1.80)	8 (1.60)	13 (2.61)	
Occasionally	8 (0.53)	4 (0.80)	2 (0.40)	2 (0.40)	
No exercise	114 (7.61)	37 (7.39)	34 (6.81)	43 (8.63)	
Smoking (%)	56 (3.74)	24 (4.79)	19 (3.81)	13 (2.61)	0.191
Alcohol use (%)	65 (4.34)	22 (4.39)	22 (4.41)	21 (4.22)	0.987
FBG (mg/dL)	90.71 ± 24.46	85.33 ± 20.02	92.27 ± 24.40	94.55 ± 27.49	<0.001
TC (mg/dL)	191.46 ± 38.91	194.01 ± 37.58	193.13 ± 39.80	187.21 ± 39.05	0.024
TG (mg/dL)	99.34 (76.28128.62)	81.60 (63.86106.44)	99.34 (79.83127.73)	116.20 (92.25141.92)	<0.001
LDL-C (mg/dL)	121.04 (97.84145.01)	116.40 (93.58142.31)	123.36 (100.93147.53)	123.17 (99.38145.40)	0.007
HDL-C (mg/dL)	54.14 (46.79 62.65)	63.03 (54.91 73.47)	53.36 (47.76 60.33)	47.56 (42.54 54.81)	0.049
WBC (×10^9/L)	6.31 ± 1.63	6.02 ± 1.54	6.46 ± 1.81	6.45 ± 1.47	<0.001
Hypertension (%)	624.00 (41.66)	181.00 (36.13)	221.00 (44.29)	222.00 (44.58)	0.009
Diabetes (%)	134.00 (8.95)	34.00 (6.79)	56.00 (11.22)	44.00 (8.84)	0.049
Antihypertensive medication (%)	528 (35)	148 (30)	184 (37)	196 (39)	0.003
Antidiabetic medication (%)	106 (7.1)	27 (5.4)	44 (8.8)	35 (7.0)	0.107

Mean ± SD for continuous variables with normal distribution, otherwise median (quartiles): the *p*-value was calculated by ANOVA (analysis of variance) or rank-sum test. N(%) for categorical variables: the *p*-value was calculated by the chi-square test. SBP, systolic blood pressure; DBP, diastolic blood pressure; WHtR, waist-height ratio; BMI, body mass index; FBG, fasting blood glucose; TC, total cholesterol; TG, total triglyceride; LDL-C, low-density lipoprotein cholesterol; HDL-C, high-density lipoprotein cholesterol; WBC, white blood cell counts; MetS –IR, metabolic score for insulin resistance.

### Association of MetS-IR and MetS risk

**Figure 2:**
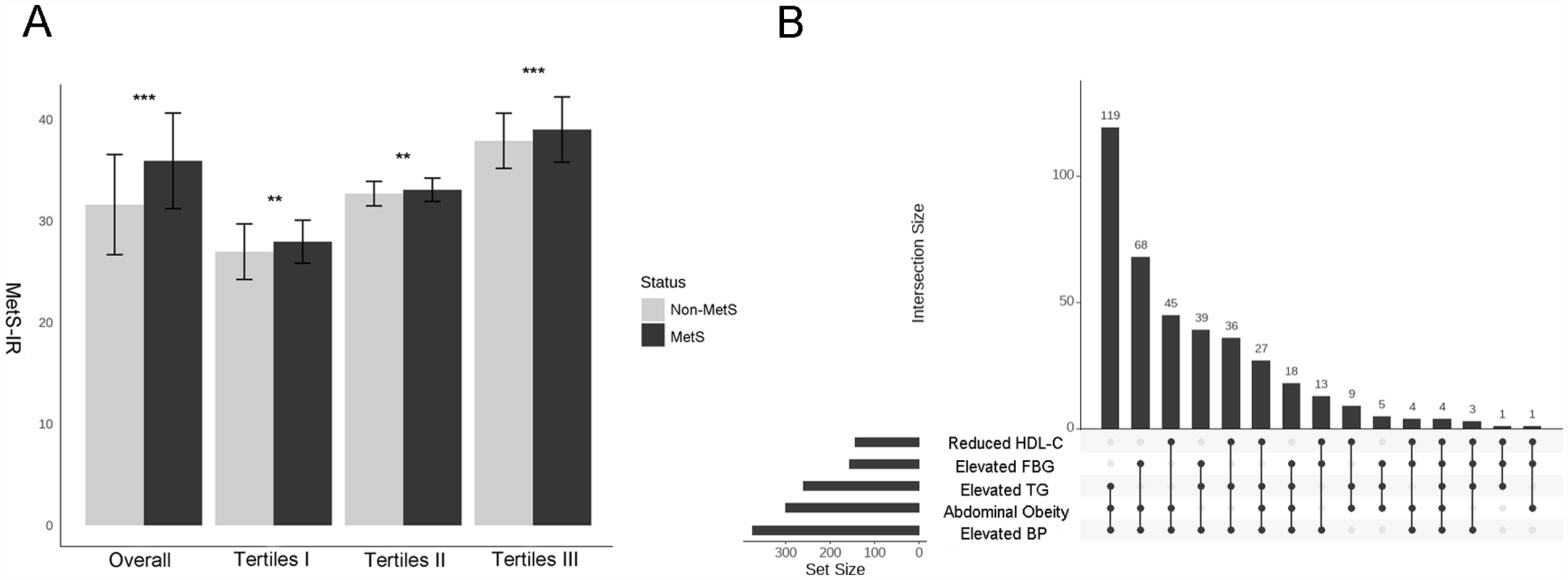
Differences in the levels of MetS–IR between the MetS and non- MetS groups and the categories of MetS. **(A)** Histograms of MetS –IR levels between Non-MetS and MetS groups under different strata, with Student’s *t*-tests used to compare the differences between the two groups. *p < 0.05, **p < 0.01, ***p < 0.001. **(B)** Upset plot shows the counts of participants with one or multiple target metabolic syndrome components. Matrix layout for all intersections of five metabolic syndrome components, sorted by intersection size. Dark circles in the matrix indicate sets that are part of the intersection. (This matrix layout shows the intersections of the five metabolic syndrome components, arranged by intersection size. Sets included in each intersection are indicated by dark circles in the matrix).

**Figure 3:**
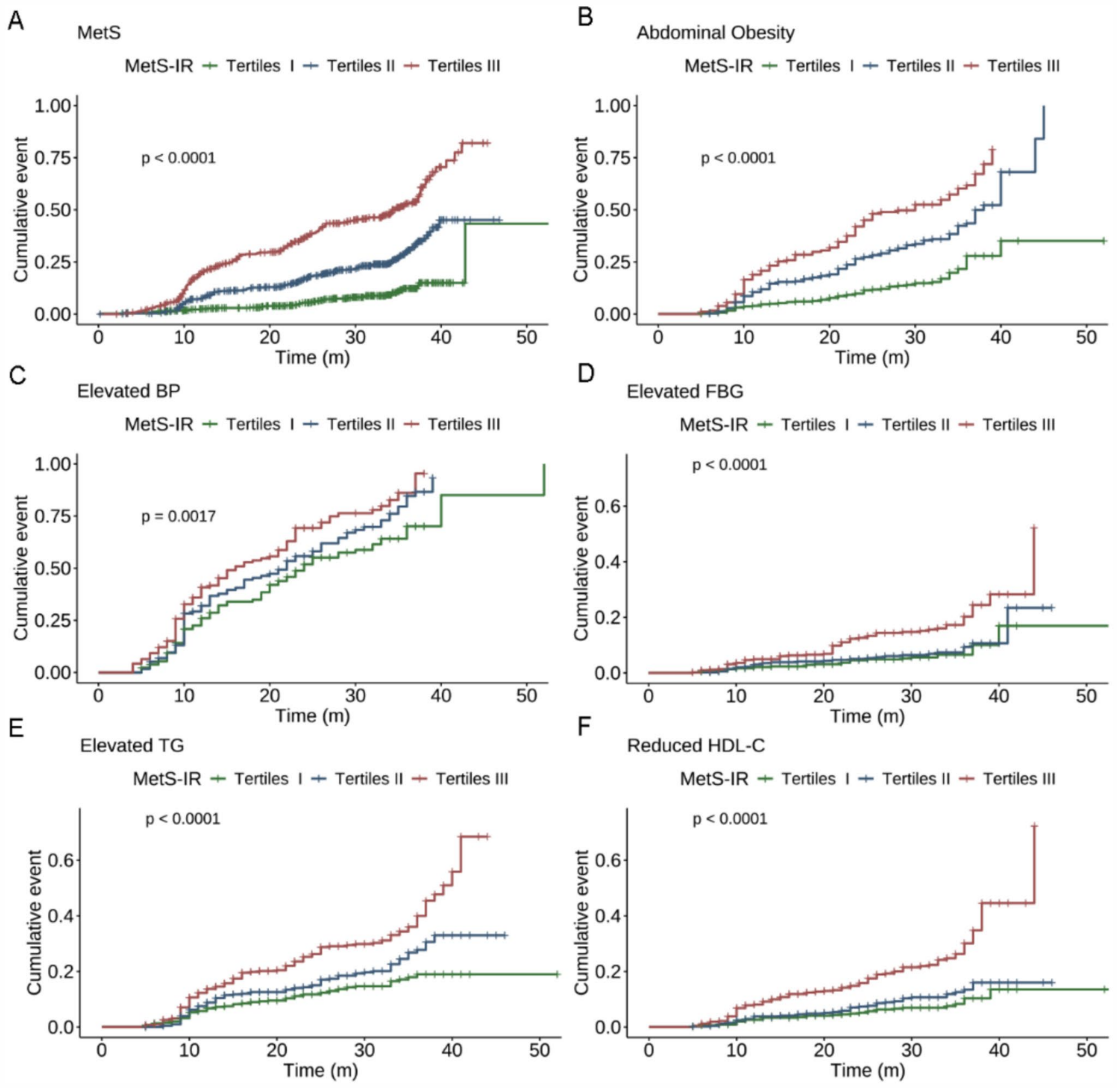
Kaplan–Meier survival curves of the cumulative incidence of MetS and its components according to the baseline MetS–IR categories. **(A)** MetS, **(B)** Abdominal Obesity, **(C)** Elevated BP, **(D)** Elevated FBG, **(E)** Elevated TG, **(F)** Reduced HDL-C. The log-rank test was used for comparisons between groups. MetS–IR, metabolic score for insulin resistance; BP, blood pressure; FBG, fasting blood glucose; TG, triglycerides; HDL-C, high-density lipoprotein cholesterol.

Compared to the subjects in the non-MetS group, higher MetS-IR was significantly detected in those in the MetS group (*p* < 0.001), and this trend remained consistent when MetS-IR was divided into three categories (Figure 2A). Our findings revealed that elevated BP accounted for the largest component of MetS, followed by central obesity; while the common combinations co-occurrences were the combinations of elevated BP, abdominal obesity and elevated TG (Figure 2B). Furthermore, Kaplan–Meier survival curve analysis demonstrated that the cumulative incidence of MetS increased with each higher MetS-IR tertile (*P_log-rank_* < 0.001, Figure 3A); Consistent trends were also observed in each component of MetS (Figures 3B–F) and in two or more MetS components (Supplementary Figure S1). As depicted in Table 2, multivariable-adjusted Cox proportional hazards regression analysis indicated that 1-SD increment of MetS-IR resulted in 1.15-fold higher risk of developing MetS (95% CI 1.13–1.18). Compared with the subjects in the first tertile of MetS-IR, the HRs of those in the second and the third tertile of MetS-IR were 2.72 (95% CI 1.92–3.85), 6.31 (95% CI 4.55–8.76), respectively; and the trend test was statistically significant (*P*_trend_ < 0.05). When further analysis of the associations between MetS-IR with the single risk factor and the number of MetS components, the increased risk of developing abdominal obesity, elevated BP, elevated FBG, elevated TG and elevated HDL-C for 1-SD increment of MetS-IR was 1.16 (95% CI 1.13–1.19), 1.04 (95% CI 1.02–1.07), 1.09 (95% CI 1.05–1.13), 1.07 (95% CI 1.05–1.10), 1.10 (95% CI 1.06–1.13), respectively. Besides, in participants with two or more MetS components, an association with MetS-IR was found in both the unadjusted and adjusted models. The adjusted HRs of the association between the MetS-IR (analyzed as continuous variables) and the cluster of MetS components (presence of two or more symptoms) were 1.06(95%CI 1.04, 1.09), 1.11(95%CI 1.08, 1.13) and 1.31(95%CI 1.22, 1.4), respectively.

**Table 2:** Univariable and multivariable Cox proportional hazards model for MetS and its components.

Outcome	Case/total	Incidence rate	Continuous		Tertiles I	Tertiles II		Tertiles III		P for trend
			HRs (95%CI)	*P*-value	HRs (95%CI)	HRs (95%CI)	*P*-value	HRs (95%CI)	*P*-value	
Model 1										
MetS	392/1498	12.21	1.16 (1.13, 1.18)	<0.001	Reference	2.93 (2.08, 4.14)	<0.001	6.76 (4.89, 9.34)	<0.001	<0.001
MetS components										
Abdominal Obesity	270/933	14.16	1.14(1.11, 1.17)	<0.001	Reference	2.39(1.77, 3.24)	<0.001	4.17(3.04, 5.73)	<0.001	0.001
Elevated BP	251/390	42.33	1.04(1.02, 1.07)	<0.001	Reference	1.33(0.97, 1.83)	0.074	1.72(1.26, 2.35)	0.001	0.001
Elevated FBG	120/1282	4.09	1.09(1.05, 1.13)	<0.001	Reference	1.16(0.69, 1.98)	0.572	2.72(1.72, 4.29)	<0.001	<0.001
Elevated TG	276/1248	10.26	1.08(1.06, 1.11)	<0.001	Reference	1.48(1.07, 2.04)	0.018	2.46(1.82, 3.33)	<0.001	<0.001
Reduced HDL-C	175/1357	5.71	1.1(1.07, 1.14)	<0.001	Reference	1.44(0.92, 2.26)	0.107	3.61(2.43, 5.36)	<0.001	<0.001
Number of MetS components										
1 vs. 0	522/659	33.96	1.02(1, 1.04)	0.077	Reference	1.03(0.85, 1.25)	0.733	1.25(0.97, 1.1.61)	0.086	0.133
2 vs. 0	447/584	33.31	1.06(1.04, 1.09)	<0.001	Reference	1.57(1.22, 2.02)	<0.001	2.04(1.59, 2.62)	<0.001	<0.001
3 vs. 0	336/473	40.00	1.11(1.09, 1.13)	<0.001	Reference	2.92(2.04, 4.18)	<0.001	4.84(3.44, 6.81)	<0.001	<0.001
≥ 4 vs. 0	56/193	14.41	1.29(1.22, 1.36)	<0.001	Reference	6.13(1.23, 30.43)	0.027	58.06(14.05, 239.86)	<0.001	<0.001
Model2										
MetS	392/1498	12.21	1.15 (1.13, 1.18)	<0.001	Reference	2.72 (1.92, 3.85)	<0.001	6.31 (4.55, 8.76)	<0.001	<0.001
MetS components										
Abdominal Obesity	270/933	14.16	1.16(1.13, 1.19)	<0.001	Reference	2.87(2.1, 3.94)	<0.001	5.27(3.75, 7.41)	<0.001	<0.001
Elevated BP	251/390	42.33	1.04(1.02, 1.07)	<0.001	Reference	1.41(1.02, 1.95)	0.039	1.7(1.23, 2.35)	0.001	0.001
Elevated FBG	120/1282	4.09	1.09(1.05, 1.13)	<0.001	Reference	1.15(0.67, 1.97)	0.622	2.79(1.75, 4.47)	<0.001	<0.001
Elevated TG	276/1248	10.26	1.07(1.05, 1.1)	<0.001	Reference	1.39(1, 1.93)	0.049	2.24(1.64, 3.04)	<0.001	<0.001
Reduced HDL-C	175/1357	5.71	1.1(1.06, 1.13)	<0.001	Reference	1.32(0.84, 2.07)	0.233	3.19(2.12, 4.78)	<0.001	<0.001
Number of MetS components										
1 vs. 0	522/659	33.96	1.01(0.99, 1.03)	0.236	Reference	0.99(0.81, 1.2)	0.883	1.16(0.88, 1.51)	0.288	0.433
2 vs. 0	447/584	33.31	1.06(1.04, 1.09)	<0.001	Reference	1.52(1.16, 1.98)	0.002	2.01(1.55, 2.61)	<0.001	<0.001
3 vs. 0	336/473	40.00	1.11(1.08, 1.13)	<0.001	Reference	2.61(1.81, 3.77)	<0.001	4.34(3.02, 6.24)	0	<0.001
≥ 4 vs. 0	56/193	14.41	1.31(1.22, 1.4)	<0.001	Reference	5.47(1.07, 28.04)	0.042	63.17(14.19, 281.13)	<0.001	<0.001

Model 1: Crude model, no covariates were adjusted. Model 2: Adjusted model, age, sex, smoking, alcohol use, exercise frequency, WBC, antihypertensive medication, and antidiabetic medication were adjusted. 95%CI, 95% confidence interval; HR, hazard ratio. incidence rate: per 100 person-years.

### Dose–response relationship between MetS-IR and MetS

**Figure 4:**
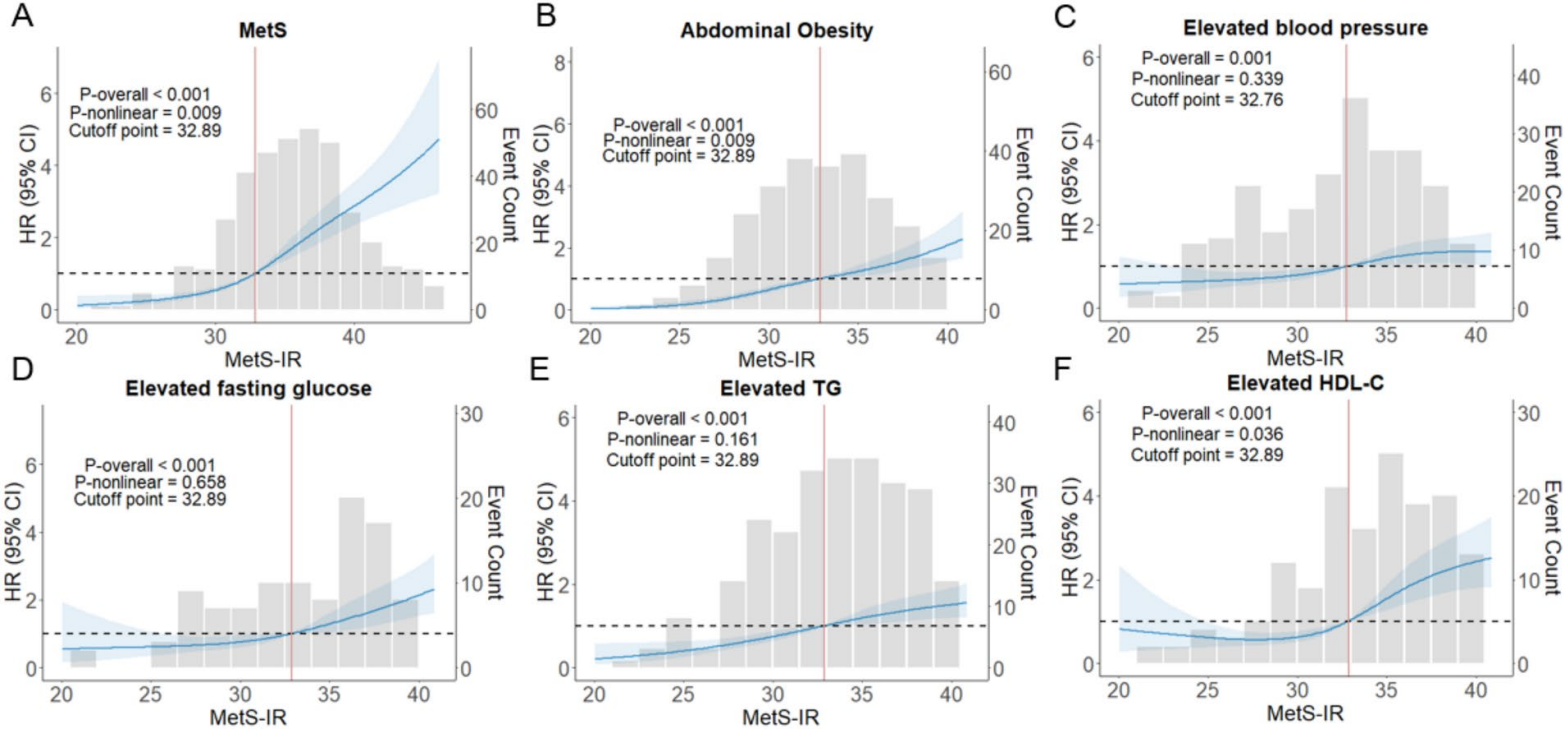
The nonlinear associations between MetS–IR and the hazard ratios of MetS and its components were analyzed using restricted cubic splines **(A–F)**. RCS with four knots were used, and the models were adjusted for age and gender. 95% CI, 95% confidence interval; HR, hazard ratio; p-overall, *p*-value for model tests; p-nonlinear, *p*-value for nonlinear tests.

Furthermore, the RCS analysis demonstrated a nonlinear dose–response relationship between MetS-IR and MetS risk (*P*_overall_ ≤ 0.001, *P*_nonlinear_ = 0.009), and the HRs of MetS increased rapidly when MetS-IR was greater than 32.89 (Figure 4A). Nevertheless, we found positive linear associations of MetS-IR with elevated BP (*P*_overall_ ≤ 0.001, *P*_nonlinear_ = 0.339), elevated FBG (*P*_overall_ ≤ 0.001, *P*_nonlinear_ = 0.658), and elevated TG (*P*_overall_ ≤ 0.001, *P*_nonlinear_ = 161) (Figures 4B–F); Interestingly, when analyzing the number of MetS components as outcomes, we found that MetS-IR was positively correlated with the presence of 2, 3, and ≥ 4 MetS components. However, a nonlinear relationship was only observed in participants with 3 or more MetS components (Supplementary Figure S2). Two-segment Cox regression analysis showed that a 1-SD increment in MetS–IR was associated with a 1.21-fold increase in the risk of higher MetS status (HR = 1.209, 95% CI: 1.109–1.319, *p* < 0.001) when MetS–IR levels were below 32.89. Similarly, a 1-SD increment in MetS–IR was associated with a 1.12-fold increase in the risk of higher MetS status (HR = 1.124, 95% CI: 1.089–1.161, *p* < 0.001) when MetS–IR levels were greater than or equal to 32.89 (Supplementary Table S1).

### Subgroup analyses and sensitivity analysis

**Figure 5:**
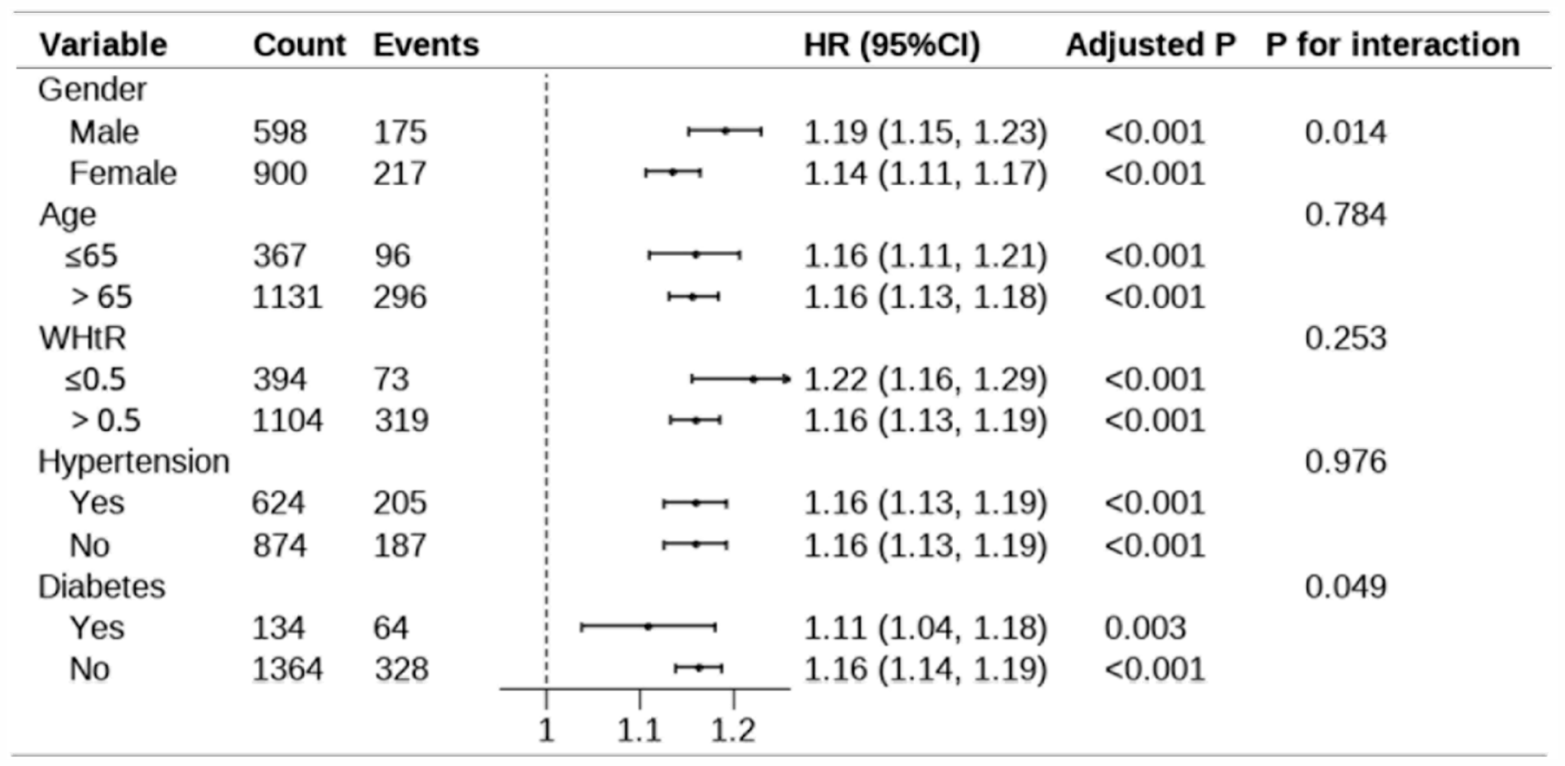
Subgroup analysis for the association between MetS-IR and MetS. The model adjust for age, sex, smoking, alcohol use, exercise frequency, WBC, antihypertensive medication, and antidiabetic medication. In the subgroup analysis stratified by gender and age, the model is not adjusted for gender and age, respectively. Adjusted *p*-values were calculated using the Benjamini post-hoc test. “Count” represents the total number of participants in each subgroup, while “Events” indicates the number of cases observed in each subgroup. The p-values for interaction assess the heterogeneity of associations across subgroups. WHtR, waist-to-height ratio; 95% CI, 95% confidence interval; HR, hazard ratio.

Further subgroup analysis was performed to explore the robustness and reliability of the relationships between MetS-IR and MetS (Figure 5). A significant positive relationship between MetS-IR and MetS was observed across different demographic settings, consistent with previous analyses of the whole population. Interaction tests revealed significant interaction effects between MetS-IR and gender, diabetes, indicating that these factors may influence the positive correlation between MetS-IR and MetS (P for interaction <0.05). Additionally, a sensitivity analysis was conducted, revealing that each standard deviation increase in MetS-IR was associated with a 16% higher risk of MetS in Model 2 (Supplementary Table S2).

### ROC analysis for predicting the incidence of MetS development

**Figure 6:**
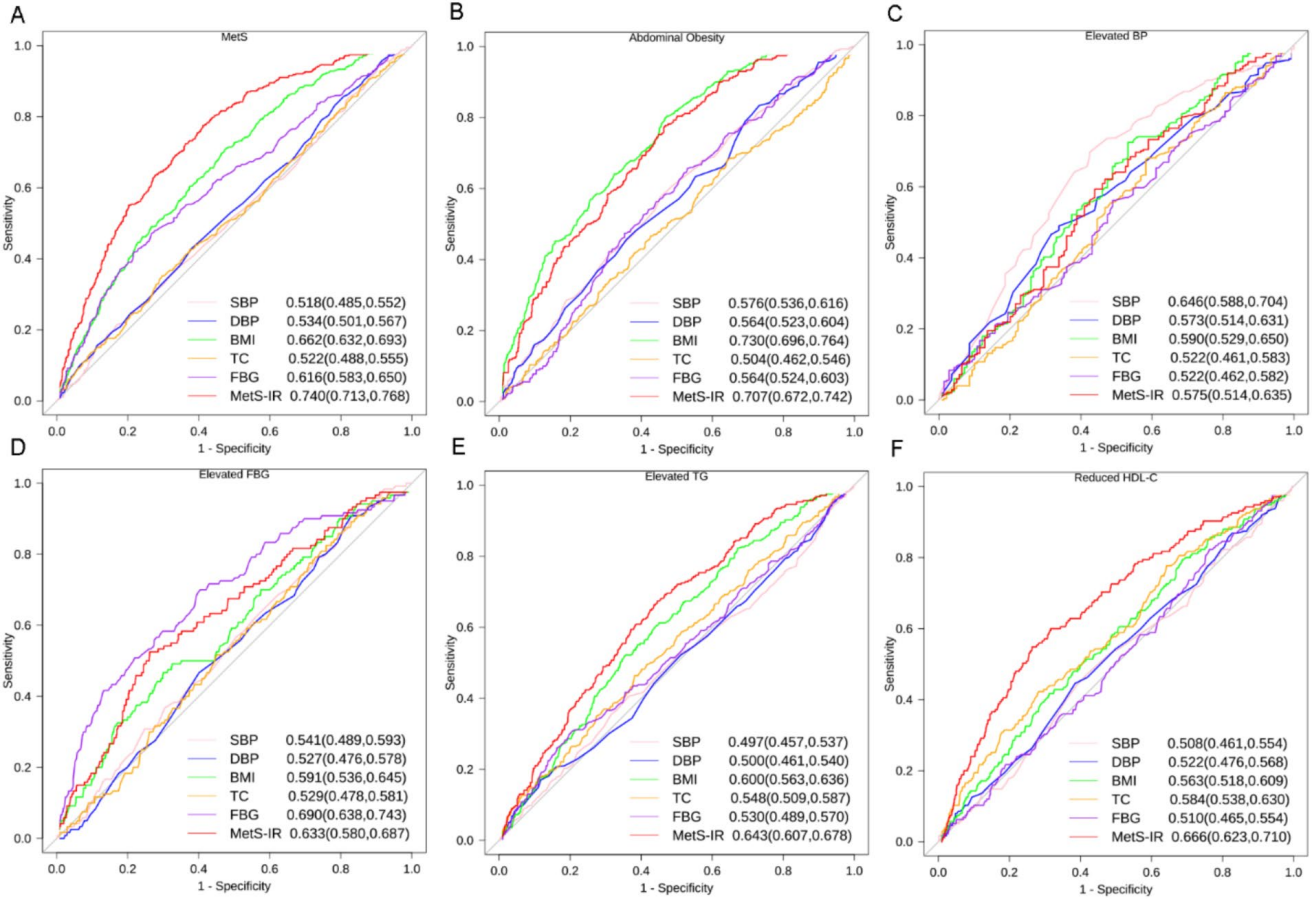
ROC curves for different indicators predicting incident MetS and its components. **(A)** MetS, **(B)** Abdominal Obesity, **(C)** Elevated BP, **(D)** Elevated FBG, **(E)** Elevated TG, **(F)** Reduced HDL-C. Footnotes are area under the curve and 95% confidence intervals. SBP, systolic blood pressure; DBP, diastolic blood pressure; BMI, body mass index; TC, total cholesterol; FBG, fasting blood glucose; MetS-IR, metabolic score for insulin resistance; TG, triglycerides; HDL-C, high-density lipoprotein cholesterol.

ROC curves were conducted to identify the optimal predictive indicator for MetS and its associated components by MetS-IR, SBP, DBP, BMI, TC, and FBG (Figure 6). The results showed that the AUC of MetS-IR, SBP, DBP, BMI, TC and FBG for MetS were 0.713 (0.74, 0.768), 0.518 (0.485, 0.552), 0.534 (0.501, 0.567), 0.662 (0.632, 0.693), 0.522 (0.488, 0.555) and 0.616 (0.583, 0.650), respectively. More importantly, the ROC curve of MetS-IR was significantly different from those of the other indicators (*p* < 0.001), suggesting a superior predictive performance for MetS. The optimal cut-off value for MetS-IR was determined to be 32.7, with the HRs being 3.92(95%CI, 3.09–4.98). As for the MetS components, MetS-IR also had the highest predictive power for elevated TG and reduced HDL-C, and exhibited significant differences with other indicators. Additionally, we found that MetS-IR had the highest AUCs in predicting the occurrence of 2, 3, and > 4 MetS components, with the values of 0.794, 0.857, and 0.931, respectively (Supplementary Figure S3).

## Discussion

In this cohort study, our results supported a positive association between MetS-IR and incident MetS and its components in the middle-aged and older adult Chinese population. This relationship exhibited a non-linear dose–response relationship between MetS-IR with incident MetS, abdominal obesity, and reduced HDL-C. A MetS-IR index exceeding 32.89 is suggestive of an elevated risk for MetS diseases. Compared with other metrics, MetS demonstrates the highest AUC, suggesting that MetS serves as a superior predictor for MetS. These findings indicate that maintaining lower MetS-IR levels might alleviate the onset of MetS among middle-aged and older adults.

MetS is widely acknowledged as a significant predictor of cardiovascular disease and cognitive impairment in the older adult population (21, 22). In our study cohort, the incidence rate of MetS was 12.21 cases per 100 person-years. Relevant studies have reported a positive association between MetS in the older adult and an increased incidence and progression of mild cognitive impairment to dementia (23). MetS is associated with various risk factors and several proposed pathophysiological mechanisms, such as insulin resistance (IR), chronic low-grade inflammation, and oxidative stress, with IR possibly being the primary cause (24–26). IR is a critical biomarker for MetS, with several key indicators including insulin (27), C-peptide (28), and the Homeostatic Model Assessment of Insulin Resistance (HOMA-IR) (29). Despite their importance, these biomarkers have notable drawbacks. Insulin and C-peptide levels lack consistent reference ranges and require specific laboratory conditions for measurement (30–32). HOMA-IR, although widely used, suffers from variable reference values and the need for precise fasting plasma insulin and glucose data, making its standardization and practical application challenging (33).

MetS-IR, a novel alternative indicator for IR, has emerged as a widely applied and promising metric, primarily utilized for cardiovascular health assessment and IR screening (13). Extensive epidemiological evidence has demonstrated that MetS-IR can be utilized to predict and assess the risk of various MetS components, including obesity (34), T2DM (35), hypertension (14), and dyslipidemia (36). Given these diverse applications, MetS-IR promises to become an important comprehensive indicator in clinical practice. The mechanisms through which IR impacts metabolic health further underscore the utility of MetS-IR as an effective measure. First, IR leads to reduced cellular uptake of glucose in the bloodstream, resulting in elevated blood glucose levels. A cohort study in China found that increased MetS-IR elevated the incidence of T2DM in the rural population (37). Second, the metabolic effects of IR, through increased sympathetic nervous system (SNS) activity and renal sodium retention, contribute to the development of hypertension (38). A retrospective study in Japan involving 15,453 participants showed that a one-unit increase in MetS-IR was associated with a 0.95-fold and 1.12-fold increase in pre-hypertension and hypertension, respectively (39). Furthermore, the body experiences elevated insulin levels under insulin-resistant conditions, exposing the liver to relative hyperinsulinemia. Insulin can inhibit the breakdown of fat in adipose tissue under catecholamine stimulation and stimulate the uptake of glucose in adipocytes for storage as fat, thereby promoting lipogenesis, leading to increased VLDL and reduced HDL particle production, manifesting as elevated plasma triglycerides, decreased HDL cholesterol, and abdominal obesity (40–42). Chinese researchers have found that MetS-IR can influence the risk of stroke through its impact on LDL-C levels (36). In summary, The various metabolic disturbances observed in MetS, including dyslipidemia, hypertension, and altered glucose metabolism, can be attributed to the abnormal physiological responses driven by elevated insulin concentrations in the insulin-resistant state.

The multivariable Cox regression and RCS analysis confirmed an overall correlation between MetS-IR and MetS. However, the RCS analysis revealed a more nuanced, non-linear relationship between these variables. Specifically, the data indicated the existence of an effect starting point, when MetS–IR < 32.89, the HR curve remains relatively stable, whereas when MetS–IR > 32.89, the HR increases significantly, indicating that 32.89 is a critical turning point for risk changes. Individuals with MetS–IR levels above this threshold are likely part of a high-risk population, necessitating closer monitoring and potential intervention. For instance, research in a Chinese population demonstrated a significant non-linear relationship between MetS-IR and the risk of prediabetes, with a clear quantitative saturation point (43). Similarly, a 10-year longitudinal study reported an early J-shaped dose–response relationship between MetS-IR and the risk of total stroke (44). Taken together, these non-linear analyses provide important insights into the complex associations between MetS-IR and various disease outcomes. By identifying critical thresholds, this approach can help elucidate the mechanistic underpinnings of MetS-IR-related pathologies. Accordingly, future research should continue to explore the non-linear relationships between MetS-IR and other health conditions, with the aim of informing more precise, evidence-based clinical decision-making.

The different effects of MetS on different gender populations can be attributed to several factors. Firstly, lifestyle-related disparities play a significant role. Males generally exhibit higher rates of smoking and alcohol consumption, as well as lower levels of physical activity, compared to females. These unhealthy lifestyle habits contribute to the accumulation of abdominal fat, which is a key component of MetS. Numerous studies have demonstrated the detrimental effects of smoking (45), alcohol consumption (46), and excessive adiposity (1) on glucose metabolism, leading to the development of IR and, consequently, an increased risk of MetS (47). Secondly, the age-related decline in testosterone levels observed in older adult men may also contribute to the gender-specific differences in MetS susceptibility. Testosterone deficiency has been linked to the development of IR and abnormal glucose metabolism, both of which are important risk factors for MetS (48–50). The decline in estrogen levels and the concurrent increase in bioavailable testosterone in postmenopausal women are key drivers of visceral adipose tissue accumulation, insulin resistance, and dyslipidemia, collectively heightening the risk of MetS (51–53). Thirdly, Significant sex-specific differences in body composition, particularly in fat and muscle distribution, play a critical role in the pathophysiology and development of MetS (54). It is important to note that the relatively small sample sizes of participants with a WHtR≤0.5 and non-diabetic individuals in the study may have introduced a certain degree of randomness and error in the estimation of hazard ratios (55). This potential limitation could have obscured any meaningful differences in the effect sizes observed between these two subgroups. Our study suggested that intervention in males may contribute to reducing incidence.

Although the AUC value of 0.740 is considered moderate, it has been regarded in the fields of epidemiology and public health as sufficient for screening high-risk populations and aiding in early risk stratification (56). For example, a study conducted in the United States reported an AUC of 0.616 for METS-IR in predicting the risk of heart failure in adults (57). Similarly, in middle-aged and older adult populations, METS-IR achieved an AUC of 0.631 for predicting hyperuricemia (58). Additionally, in a 10-year longitudinal study, the AUC of METS-IR for predicting coronary artery disease ranged between 0.53 and 0.61 (59). While these findings suggest that METS-IR has certain limitations in its predictive performance, they also highlight its potential and practical utility in assessing the risk of metabolic diseases. Furthermore, the AUC value of 0.740 observed in our study is relatively higher compared to similar studies, which may indicate its applicability in risk assessment. As older adult individuals age, the progressive decline in muscle mass is accompanied by an increase in fat proportion, despite stable overall body weight, rendering BMI an inadequate measure of metabolic risk (60, 61). Height can be significantly reduced due to spinal shortening caused by degenerative bone diseases or kyphosis, introducing additional inaccuracies into BMI calculations for older adult individuals. Although BMI is generally effective in evaluating overall obesity, its predictive capacity for central obesity and metabolic risk remains relatively limited (62–64). In contrast, METS-IR is comprised of parameters that span multiple dimensions of lipid metabolism, glucose metabolism, and body composition, thereby exhibiting a more robust association with metabolic diseases.

Several specific strengths emerged in our study. First, to our knowledge, this is the first cohort study to examine the exploration of the relationship between MetS -IR and MetS in middle-aged and older adults. This study adopts a cohort design utilizing real-world data, which provides a robust framework for establishing the causal relationship between MetS-IR and MetS. Moreover, the analytical approach accounted for a comprehensive set of potential confounding factors, thereby mitigating the risk of biased estimates. However, certain limitations are also present. Firstly, as a retrospective cohort study, this research inevitably encountered instances of missing data. To address this issue, multiple chained imputation methods were employed, revealing no significant differences in the baseline characteristics of the population before and after imputation. Furthermore, analyses performed after excluding individuals with missing data yielded results consistent with those derived from the complete dataset, thereby minimizing the potential bias associated with missing data. Secondly, although this study adjusted for various factors such as demographic characteristics, lifestyle, and medication use, residual confounding factors, including socioeconomic status, dietary habits, and diabetes duration, were not accounted for. Given that the study population was sourced from a single region, the variability in dietary patterns is likely minimal. Future research should incorporate more comprehensive data on lifestyle and socioeconomic variables to enhance the control of potential confounding biases. Thirdly, the relatively limited follow-up duration precluded the observation of cardiovascular events associated with metabolic syndrome. As this cohort forms part of a long-term public health initiative, future extended follow-up, coupled with a more comprehensive examination of contextual factors, has the potential to yield more robust evidence elucidating the relationship between MetS-IR and the risk of metabolic syndrome onset, while simultaneously offering critical insights into the progression of adverse cardiovascular outcomes. Finally, this study was conducted within an older adult population in Guangxi, China, where dietary patterns, genetic background, lifestyle factors, and metabolic risk profiles may differ from those observed in other populations. As a result, the generalizability of the findings might be constrained. To further enhance the universality of the conclusions, validation using multicenter data or more diverse populations is warranted in future research.

## Conclusion

In summary, our findings indicate a nonlinear dose–response relationship between MetS-IR and incident MetS, abdominal obesity, and reduced HDL-C in the middle-aged older adult population but linear associations of MetS-IR and elevated BP, elevated FBG, elevated TG. These observations underscore the potential clinical utility of minimizing MetS-IR as an important and effective measure to prevent MetS. The insights gained from this research provide valuable evidence to inform the development of targeted prevention strategies for MetS in middle-aged and older adult population.

## Data Availability

Due to ethical and privacy concerns, the data are not publicly available but can be requested from the corresponding author.
