# Supplementary material for: Association of metabolic score for insulin resistance with incident metabolic syndrome: a cohort study in middle-aged and older adult Chinese population
Source: Front Public Health. 2025 Feb 18;13:1453144. doi: 10.3389/fpubh.2025.1453144 (PMC11883690; doi:10.3389/fpubh.2025.1453144)
Supplement: Supplementary file 1 [file Table_1.docx]

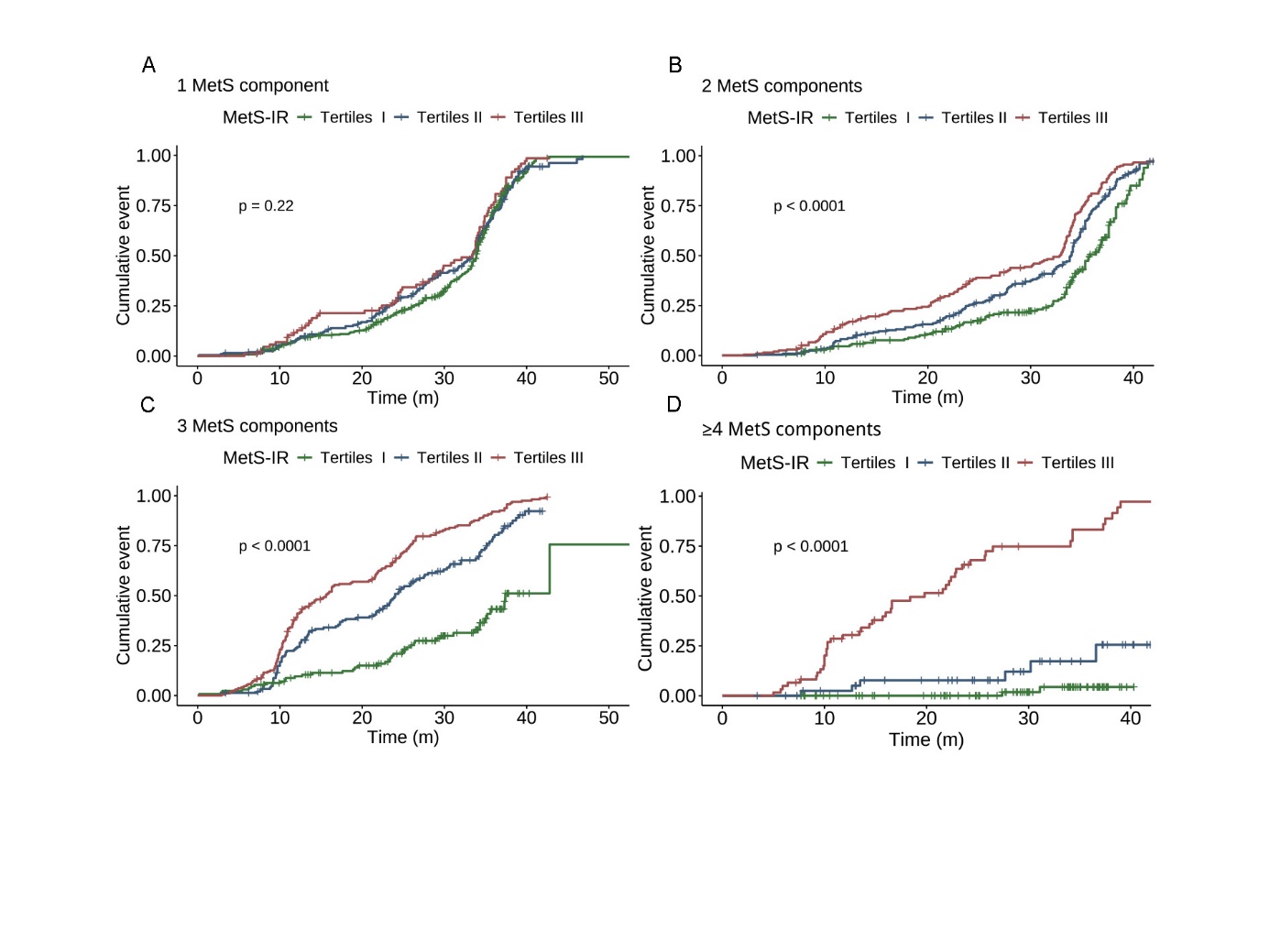


Supplementary Figure S1: Kaplan-Meier survival curves of the cumulative incidence of MetS components according to the baseline MetS–IR categories. (A) 1 MetS component, (B) 2 MetS components, (C) 3 MetS components, (D) ≥4 MetS components. MetS–IR, metabolic score for insulin resistance; p-values are calculated using the log-rank test for comparisons between groups.


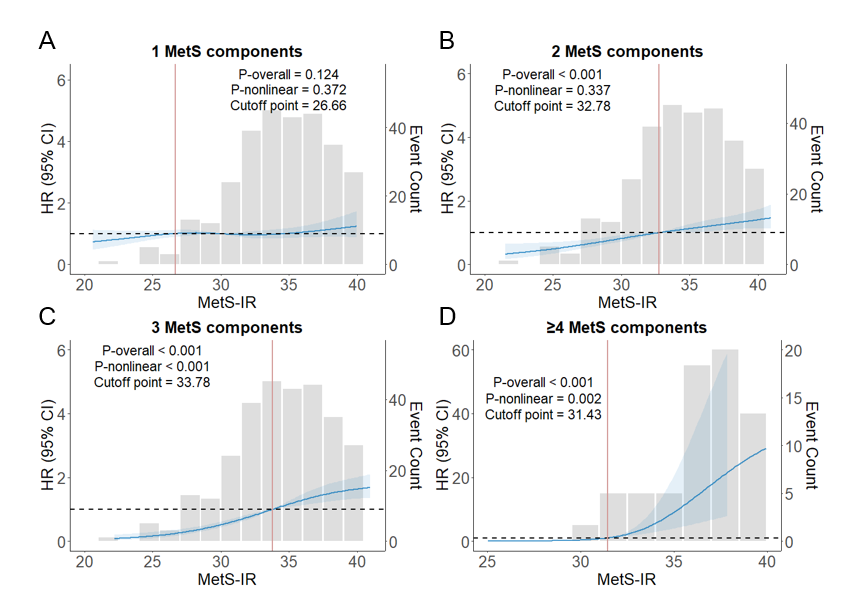


Supplementary Figure S2: The nonlinear associations between MetS–IR and the Hazard ratios of the number of MetS components using restricted cubic splines analysis. Adjusted for age, and gender.


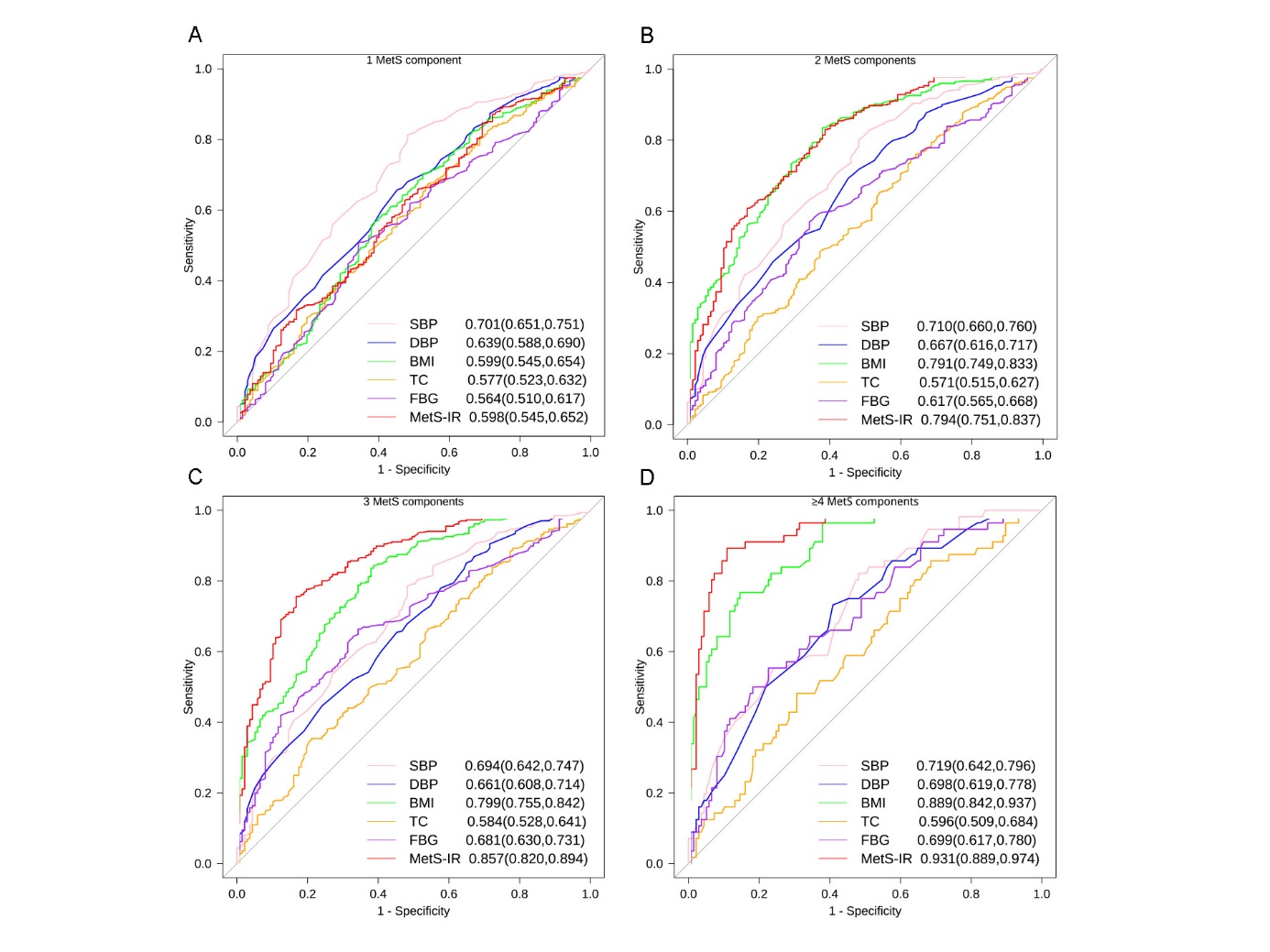


Supplementary Figure S3: ROC curves for different indicators predicting incident MetS and the number of MetS components. (A) 1 MetS component, (B) 2 MetS components, (C) 3 MetS components, (D) ≥4 MetS components. The area under the curve (AUC) and 95% confidence intervals are provided for each indicator. MetS–IR, metabolic score for insulin resistance; SBP, systolic blood pressure; DBP, diastolic blood pressure; BMI, body mass index; TC, total cholesterol; FBG, fasting blood glucose.

Supplementary Table S1: Threshold effect analyses of MetS-IR and the risk of MetS-IR and the risk of MetS using two-piecewise Cox proportional hazards models.

| Group | n | Events | HR (95%CI) | Z | P |
| --- | --- | --- | --- | --- | --- |
| MetS-IR<32.89 | 765 | 97 | 1.21(1.11,1.32) | 4.288 | <0.001 |
| MetS-IR≥32.89 | 733 | 295 | 1.12(1.09,1.16) | 7.234 | <0.001 |

The model adjusted for age, sex, smoking, alcohol use, exercise frequency, WBC, antihypertensive medication, and antidiabetic medication.

Supplementary Table S2: Sensitivity analysis of univariable and multivariable Cox proportional hazards models assessing the association between MetS-IR and the risk of MetS.

| Outcome | case/total | incidence rate | continuous | | Tertiles I | Tertiles II | | Tertiles III | | P for trend |
| --- | --- | --- | --- | --- | --- | --- | --- | --- | --- | --- |
|  |  |  | HR (95%CI) | P value | HR (95%CI) | HR (95%CI) | P value | HR (95%CI) | P value |  |
| Model 1 |  |  |  |  |  |  |  |  |  |  |
| MetS | 337/1300 | 11.93 | 1.16 (1.13,1.18) | <0.001 | Reference | 3.02 (2.10, 4.34) | <0.001 | 6.75 (4.79,9.50) | <0.001 | <0.001 |
| Model 2 |  |  |  |  |  |  |  |  |  |  |
| MetS | 337/1300 | 11.93 | 1.16 (1.13,1.18) | <0.001 | Reference | 2.68 (1.85,3.85) | <0.001 | 6.19 (4.38,8.74) | <0.001 | <0.001 |

After excluding individuals with imputed data.

Model 1: Crude model, no covariates were adjusted.

Model 2: Adjusted model, age, sex, smoking, alcohol use, exercise frequency, WBC, antihypertensive medication, and antidiabetic medication were adjusted.

95%CI, 95% confidence interval; HR, hazard ratio.

incidence rate: per 100 person-years
